# Deep sequencing-based analysis of the anaerobic stimulon in *Neisseria gonorrhoeae*

**DOI:** 10.1186/1471-2164-12-51

**Published:** 2011-01-20

**Authors:** Vincent M Isabella, Virginia L Clark

**Affiliations:** 1 Department of Microbiology and Immunology, School of Medicine and Dentistry, University of Rochester, Box 672, 601 Elmwood Avenue, Rochester, NY 14642, USA

## Abstract

**Background:**

Maintenance of an anaerobic denitrification system in the obligate human pathogen, *Neisseria gonorrhoeae*, suggests that an anaerobic lifestyle may be important during the course of infection. Furthermore, mounting evidence suggests that reduction of host-produced nitric oxide has several immunomodulary effects on the host. However, at this point there have been no studies analyzing the complete gonococcal transcriptome response to anaerobiosis. Here we performed deep sequencing to compare the gonococcal transcriptomes of aerobically and anaerobically grown cells. Using the information derived from this sequencing, we discuss the implications of the robust transcriptional response to anaerobic growth.

**Results:**

We determined that 198 chromosomal genes were differentially expressed (~10% of the genome) in response to anaerobic conditions. We also observed a large induction of genes encoded within the cryptic plasmid, pJD1. Validation of RNA-seq data using translational-*lacZ *fusions or RT-PCR demonstrated the RNA-seq results to be very reproducible. Surprisingly, many genes of prophage origin were induced anaerobically, as well as several transcriptional regulators previously unknown to be involved in anaerobic growth. We also confirmed expression and regulation of a small RNA, likely a functional equivalent of *fnrS *in the *Enterobacteriaceae *family. We also determined that many genes found to be responsive to anaerobiosis have also been shown to be responsive to iron and/or oxidative stress.

**Conclusions:**

Gonococci will be subject to many forms of environmental stress, including oxygen-limitation, during the course of infection. Here we determined that the anaerobic stimulon in gonococci was larger than previous studies would suggest. Many new targets for future research have been uncovered, and the results derived from this study may have helped to elucidate factors or mechanisms of virulence that may have otherwise been overlooked.

## Background

*N. gonorrhoeae *was long considered to be an obligate aerobe until it was discovered that anaerobic growth was possible when nitrite or nitric oxide (NO) was used as a terminal electron acceptor [1,2]. Anaerobic growth is accomplished through utilization of a truncated denitrification pathway, which is encoded within the gonococcal genome as a pair of divergently transcribed genes, *aniA*, encoding a nitrite reductase, and *norB*, encoding a nitric oxide reductase [3,4]. Anaerobiosis is presumed to be a physiologically significant state during infection, as the gonococcus is often recovered from infected individuals in co-culture with obligate anaerobes such as *Peptococcus *and *Bacteroides *spp. [5]. Furthermore, gonococci have been shown to induce and repress the expression of several genes in response to anaerobiosis, and antibody to AniA, the major anaerobically induced outer membrane protein, can be found in sera from infected women, demonstrating that this protein is expressed *in vivo *[6,7].

The ability of gonococci to utilize this denitrification pathway to reduce NO may have immunomodulary effects during the course of infection. Some evidence suggests that the gonococcal reduction of host-produced NO may be responsible, at least in part, for the high incidence of asymptomatic disease [4,8,9]. *In vitro*, *N. gonorrhoeae *was shown to be capable of setting a NO steady-state level in the anti-inflammatory range [8]. Recently, in an *in vitro *cervical cell model of infection, it was shown that gonococcal activation of iNOS promoted bacterial survival. In this study, it was suggested that host-derived nitric oxide is not protective against gonococci, rather, nitric oxide may actually be required to sustain cervical bacterial disease [10].

Previous research has shown that the genes involved in denitrification and/or adaptation to anaerobic growth in *Neisseria *spp. are subject to transcriptional regulation by the oxygen-sensitive regulator, FNR, the NO-sensitive repressor, NsrR, and the nitrite-insensitive two component system, NarQP [3,4,7,11,12]. Earlier data from a microarray-based approach suggested that the gonococcal FNR-regulon was composed of fourteen activated and six repressed transcripts, making the gonococcal FNR regulon much smaller than that of *E. coli*, where FNR was shown to regulate more than 100 operons [7,13]. Discovery of NsrR and NarQP-regulated genes in *Neisseria *spp. has relied mainly on *in silico *analysis of intergenic chromosomal regions to identify similarity to previously defined regulator binding sites. As is the case with FNR, the neisserial NsrR and NarQP regulons, at least the currently defined members, are much smaller than their *E. coli *counterparts [3,12,14,15].

In this study we employ a powerful whole-genome approach, RNA-seq, to quantitatively sequence the complete gonococcal transcriptome. Using this method we were able to define global changes in gene expression in response to anaerobiosis. We show that the gonococcal anaerobic stimulon is not small, and that 198 chromosomal open reading frames (~10% of the genome) are differentially expressed. We present several novel findings that, taken together, support the view that anaerobic growth is an important facet of life for this organism, and should be considered when studying the host/pathogen interaction.

## Results and Discussion

### Sequencing the gonococcal transcriptome

**Figure 1:**
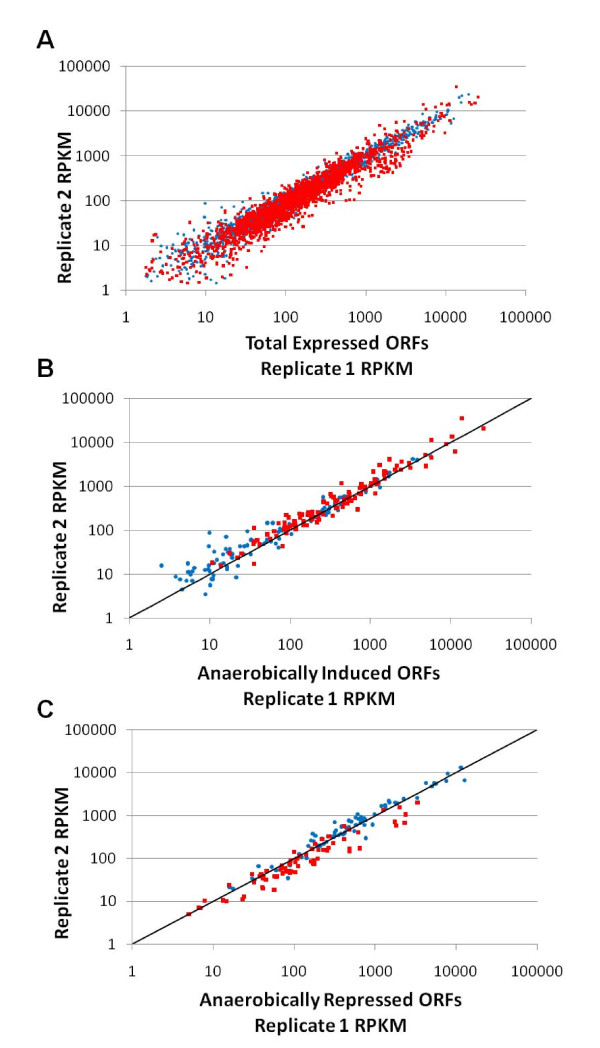
**Reproducibility of expression level between replicates**. (A) The RPKM of all genes with non-zero expression, (B) anaerobically induced, and (C) anaerobically repressed genes were plotted, with the RPKM of replicate 1 plotted on the x-axis, and the RPKM of replicate 2 plotted on the y-axis. Each spot represents a single gene. Blue circles represent genes expressed aerobically, and red squares represent genes expressed anaerobically. For (B) and (C) a line of *m *= 1 is included in the chart area.

Whole genome mRNA sequencing is an attractive method of monitoring global changes in gene expression while overcoming many of the pitfalls of traditional DNA microarrays [16]. For the purposes of this study, anaerobic conditions were defined as anoxia, the presence of nitrite (an electron acceptor required for anaerobic growth) and the concomitant presence of nitric oxide (the product of nitrite reduction). In order to define the gonococcal anaerobic stimulon, RNA-seq was performed on two biological replicates of aerobically or anaerobically plate-grown gonococci using the ABI SOLiD™ system (see methods). Unique sequence reads from the RNA-seq data were mapped to the annotated FA1090 genome, and gene expression was quantified as reads per kilobase of coding sequence per million reads (RPKM) (See additional file 1: Supplementary Table S1). When expression data for each replicate were plotted against each other, RPKM values were observed to be adequately reproducible (Figure 1A), though expectedly, the extent of reproducibility was slightly less in genes with low expression [17]. For this reason, we required mapped genes to have an RPKM ≥ 10 in order to be considered as a candidate for differential expression. However, genes that had an RPMK < 10 under one growth condition, but were highly induced in the other condition were accepted as candidates. Genes were considered to be differentially expressed if there was a three-fold or greater difference in RPKM between the two growth conditions.

Analysis of RNA-seq data revealed that 198 chromosomal genes met the criteria for being differentially expressed, with 117 genes being anaerobically induced and 81 genes being anaerobically repressed (Table 1). Figure 1B and 1C illustrate a reasonable measure of reproducibility in expression level between replicates in the 198 differentially expressed genes.

**Table 1:** Chromosomal genes differentially expressed in response to anaerobic conditions*^a^*

Gene	ORF ID	**Rep 1**^***b***^	Rep 2	**Definition**^***c***^	**Regulon**^***d***^
*Small molecule biosynthesis*					
*hda*	NGO0187	4.1	4.1	Histone deacetylase-like protein/acetoin utilization protein	
*pdxH*	NGO0658	4.3	5.5	Pyridoxamine 5-phosphate oxidase	MtrR
*mobA*	NGO0754	3.5	4.6	Molybdopterin-guanine dinucleotide biosynthesis protein	NsrR
*leu1*	NGO0848	6.6	4.5	2-isopropylmalate synthase	
*folA*	NGO1694	3.8	2.8	Dihydrofolate reductase	RpoH
					
*Transport and binding proteins*					
*fHBP*	NGO0033	3.5	4.2	Factor H binding protein	
*hecA*	NGO0706	3.2	4.9	Filamentous hemagglutinin	
*bfrA*	NGO0794	14.1	9.9	Bacterioferritin A	
*bfrB*	NGO0795	9.7	10.1	Bacterioferritin B	MtrR
*pil*	NGO1177	3.5	3.5	Neisseria-specific type IV pilin-related protein	
	NGO1440	4.4	2.8	ABC-type amino acid transporter, periplasmic protein	
*lecA*	NGO1981	5.0	3.9	Adhesin protein	Lrp
					
*Energy metabolism/Oxidation-Reduction*					
*cybP*	NGO0805	3.2	2.8	Nickel-dependent hydrogenase, b-type cytochrome subunit	
*npd*	NGO1024	9.6	7.9	2-Nitropropane dioxygenase-like	MtrR
*aniA*	NGO1276	28.4	28.0	Nitrite reductase	NsrR, FNR
*eftD*	NGO1396	5.2	5.1	Electron transfer flavoprotein-ubiquinone oxidoreductase	
					
*Macromolecular biosynthesis*					
*rl34*	NGO2182	3.0	3. 1	50S ribosomal protein L34	
					
*Regulation*					
*farR*	NGO0058	4.0	3.7	MarR family transcriptional regulator	Lrp, MtrR
*nmlR*	NGO0602	7.2	7.6	Transcriptional regulator *nmlR*	NmlR, FNR
*xre*	NGO0797	3.2	3.4	XRE family transcriptional regulator	
*marR*	NGO1244	5.4	15.2	MarR-family transcriptional regulator	RpoH, MtrR
*nosR^a^*	NGO1401/1402	9.4	9.5	Regulator of nitrous oxide reductase pseudogenes	FNR
*lexA*	NGO1427	4.2	5.3	LexA-family repressor	LexA
*arsR*	NGO1562	6.1	2.2	ArsR-family transcriptional regulator	
					
*Cell Structure*					
*ompA*	NGO1559	3.7	3.4	Outer membrane protein	
*lpcA*	NGO1986	4.2	6.1	Phosphoheptose isomerase	
					
*Adaptation and stress response*					
*grx3*	NGO0114	3.3	6.6	Glutaredoxin 3	Fur
*recN*	NGO0318	4.7	5.5	Repair protein RecN	Fur, LexA
*htpX*	NGO0399	11.8	8.6	Heat shock protein, Peptidase family M48	Lrp
*dnrN*	NGO0653	8.9	12.3	Iron-sulfur cluster repair protein	NsrR, FNR
	NGO0757	2.6	4.1	Putative periplasmic Cpx-family sensor kinase involved in P pilus formation	
*hslR*	NGO0822	2.7	5.0	Heat shock protein HSP-15	
	NGO1022	3.8	3.4	DEDDh family exonuclease	
*clpB*	NGO1046	4.9	9.4	Endopeptidase Clp ATP-binding chain B (HSP- F84.1)	RpoH, MtrR
*recX*	NGO1053	3.6	2.8	RecX-family regulator of RecA function	
*tehB*	NGO1161	2.6	4.0	Tellurite resistance protein	
*hsp33*	NGO1189	6.1	5.2	Heat shock protein HSP-33 chaperonin	MtrR
	NGO1245	4.0	8.5	ABC-type multidrug transport system, ATPase/permease	
*hit*	NGO1273	2.3	5.5	Protein Kinase C Interacting protein belonging to the ubiquitous HIT family of hydrolases that act on alpha-phosphates of ribonucleotides	
*nudH*	NGO1334	3.3	3.3	Nudix hydrolase, dinucleoside polyphosphate hydrolase	
*grpE*	NGO1422	3.6	2.7	Heat shock protein (HSP-70 cofactor; nucleotide exchange factor)	RpoH, MtrR
*erpA*	NGO1426	2.8	2.6	Putative iron-sulfur cluster insertion protein	RpoH, Lrp
*dnaK*	NGO1429	2.7	3.4	Heat shock protein, chaperone	RpoH
	NGO1566	3.4	3.1	Nudix hydrolase, catalyzing the hydrolysis of nucleoside diphosphates	
*ntrA*	NGO1702	3.5	20.1	Nitroreductase-like family containing uncharacterized proteins similar to nitroreductase	
*ccp*	NGO1769	5.6	12.1	Cytochrome c peroxidase	
*yhhF*	NGO1860	3.7	4.1	Probable DNA methylase	
*dnaJ*	NGO1901	2.8	5.1	Heat shock protein HSP-40	RpoH
					
*Hypothetical or Unassigned*					
	NGO0010	5.1	7.2	Neisseria-specific protein, uncharacterized	
	NGO0011	3.2	3.0	Conserved hypothetical protein	
	NGO0119	5.4	5.1	Neisseria-specific protein, uncharacterized; possible ribonuclease	
	NGO0165	3.2	5.9	Neisseria-specific protein, uncharacterized	
	NGO0569	3.8	2.1	Conserved hypothetical protein (possible transcriptional regulator)	
	NGO0570	3.5	3.4	Possible DNA-binding CreA protein	RpoH
	NGO0618	3.3	2.7	Neisseria-specific protein, uncharacterized	
	DUF331	4.1	7.8	Uncharacterized protein family DUF331; the function of this family is unknown	
	NGO0802	3.7	4.0	Hypothetical protein (possible Neisseria-specific protein)	
	NGO0854	3.6	8.4	Hypothetical protein	
	NGO0895	6.4	4.6	Neisseria-specific protein, uncharacterized	
	NGO0896	4.1	5.0	Hypothetical protein	
	NGO0995	3.1	3.3	Neisseria-specific protein, uncharacterized	
	NGO1033	4.3	6.8	Probable transglycosylase	
					
	NGO1037	4.4	5.2	Hypothetical protein	
	NGO1191	3.5	2.7	Neisseria-specific protein, uncharacterized	
	NGO1277	6.6	7.0	Conserved hypothetical protein (NirV) FGE- sulfatase super family.	
	NGO1428	14.3	8.4	Neisseria-specific protein, uncharacterized	LexA
	NGO1517	4.8	4.8	Neisseria-specific protein, uncharacterized	NsrR
	NGO1793	6.4	4.3	Conserved hypothetical protein (probable integral membrane protein)	
	NGO1987	3.1	4.4	Uncharacterized protein family UPF0102; the function of this family is unknown	
	NGO2023	2.4	7.3	Conserved hypothetical protein	
					
*Small RNA fnrS^c^*		24.9	17.8	Probable small regulatory RNA	FNR
					

**Gene**	**ORF ID**	**Rep 1**	**Rep 2**	**Definition**	**Regulon**

					
*Ngo *Φ *1 phage*					
	NGO0463	9.2	5.9	Putative phage associated protein	
	NGO0464	12.3	9.0	Putative phage associated protein	
	NGO0465	15.4	7.3	Putative phage associated protein	
	NGO0467	23.6	29.8	Putative phage associated protein	
	NGO0472	94.6	19.7	Putative phage associated protein	FNR
*dnaB*	NGO0485	6.1	4.2	Replicative DNA helicase	
	NGO0488	4.2	10.0	Putative phage associated protein	
	NGO0489	10.2	16.9	Phage Holliday junction resolvase (RusA-like) protein	
	NGO0491	14.2	5.1	Putative phage associated protein	
	NGO0492	8.7	6.6	Putative phage associated protein	
	NGO0494	10.0	10.9	Putative phage associated protein	
	NGO0495	9.6	7.6	Putative phage associated protein	
	NGO0496	16.1	12.9	Putative phage associated protein	
	NGO0497	24.1	17.0	Putative phage associated protein	
	NGO0498	18.0	9.5	Putative phage associated protein	
	NGO0499	17.4	12.9	Putative phage associated protein	
	NGO0500	17.6	6.6	Putative phage associated protein	
	NGO0501	27.4	9.1	Putative phage associated protein	
	NGO0502	47.2	6.8	Putative phage associated protein	
	NGO0503	6.0	4.8	Putative phage associated protein	
	NGO0504	14.4	5.2	Putative phage associated protein	
	NGO0506	15.9	11.3	Putative phage associated protein	
	NGO0507	22.3	12.1	Putative phage associated protein	
	NGO0508	15.6	11.1	Putative phage associated protein	
	NGO0509	3.5	3.6	Putative phage associated protein	
	NGO0510	7.1	5.6	Putative phage associated protein	
	NGO0512	9.4	13.2	Putative phage associated protein	
	NGO0513	4.9	5.2	Putative phage associated protein	
	NGO0514	9.8	6.2	Putative phage associated protein	
	NGO0522	9.4	5.6	Putative phage associated protein	
					
*Ngo *Φ2 *phage*					
	NGO1100	4.1	5.0	Putative phage associated protein	
	NGO1120	75.5	16.0	Putative phage associated protein	
	NGO1131	19.4	16.8	Putative phage associated protein	
	NGO1132	11.3	16.2	Putative phage associated protein	
					
*Ngo *Φ3 *phage*					
	NGO1614	8.2	11.0	Putative phage associated protein	
	NGO1615	10.3	7.2	Conserved hypothetical protein (type I restriction enzyme related)	FNR
	NGO1624	34.0	16.0	Putative phage associated protein	
	NGO1627	6.6	4.4	Conserved hypothetical protein (probable phage origin)	
	NGO1628	7.6	4.9	Putative phage associated protein	
	NGO1633	26.9	137.3	Putative phage associated protein	
	NGO1634	27.4	18.8	Putative phage associated protein	
	NGO1635	58.1	5.4	Putative phage associated protein	
	NGO1636	26.4	10.0	Putative phage associated protein	
	NGO1637	16.6	5.5	Putative phage associated protein	
	NGO1640	22.4	10.6	Putative phage associated protein	
					
*Ngo *Φ5 *phage*					
	NGO0731	5.1	7.3	Putative phage associated protein	
	NGO0732	3.3	2.2	Putative phage associated protein	
					

**Gene**	**ID**	**Rep 1**	**Rep 2**	**Definition**	**Regulon**

					
*Small molecule biosynthesis*					
*cysK*	NGO0340	-2.8	-6.7	Cysteine synthase	FNR
*hesB*	NGO0632	-3.8	-4.0	Iron-sulfur cluster biosynthesis	
*iscU*	NGO0633	-4.8	-5.3	Iron-sulfur cluster assembly scaffold protein	
					
*iscS*	NGO0636	-4.8	-5.6	Cysteine desulferase	
*hisD*	NGO1240	-3.3	-3.2	Histidinol dehydrogenase	
*pgsA*	NGO1247	-3.8	-7.1	CDP-alcohol phosphatidyltransferase	
*asd*	NGO1997	-3.3	-5.0	Aspartate-semialdehyde dehydrogenase	
					
*Transport and binding proteins*					
*gluP*	NGO0142	-3.1	-4.3	Glucose/galactose transporter	
*nhaC*	NGO0143	-2.8	-3.3	Na+/H+ antiporter (NhaC)	
*amtB*	NGO0198	-3.7	-5.3	Ammonium Transporter Family	
*fbp*	NGO0217	-4.8	-5.6	ABC-type iron transporter, periplasmic binding protein	Fur, Lrp
*oxiA*	NGO0372	-6.7	-20.0	Bacterial extracellular solute-binding protein	FarR
	NGO0373	-3.7	-11.1	ABC-type arginine transport system, permease component	FarR
*glnQ*	NGO0374	-2.9	-11.1	ABC-type amino acid transporter, ATP-binding protein	FarR, FNR
*citT*	NGO0377	-5.6	-9.1	Di- and tri-carboxylate transporters (inorganic ion transport)	
*cysA*	NGO0445	-5.6	-9.1	ABC-type sulfate transporter, ATP-binding protein	Lrp
*sbp*	NGO0877	-4.8	-25.0	ABC-related sulfate-binding protein	
	NGO1290	-3.8	-3.3	Putative sodium/alanine symport protein	
*hemO*	NGO1318	-7.1	-12.5	Heme oxygenase/iron starvation protein	Fur
*lldP*	NGO1361	-9.1	-8.3	L-lactate permease (partial only)	
*exbD*	NGO1377	-3.4	-3.6	Membrane bound biopolymer transport protein ExbD/TolR	
*exbB*	NGO1378	-3.1	-5.0	Transport protein	
*lctP*	NGO1449	-4.0	-14.3	L-lactate permease	
*potF*	NGO1494	-7.7	-20.0	ABC-type spermidine/putrescine transporter, solute binding protein	MtrR
*putP*	NGO1552	-5.6	-11.1	Sodium/proline symport protein	
*ompU*	NGO1688	-2.6	-3.7	Putative iron uptake protein	FNR
*yaaJ*	NGO1787	-3.3	-4.3	Sodium/alanine symport protein	
	NGO1807	-2.7	-3.8	Amino-acid symport protein	
*gltS*	NGO1890	-3.1	-5.0	Sodium/glutamate symport protein	MtrR
	NGO1954	-3.6	-6.3	Di- and tri-peptide permease, PRT2	
*sstT*	NGO1957	-4.5	-11.1	Sodium/serine symport protein	
*glnM*	NGO2011	-6.7	-12.5	ABC-type amino acid transport system, permease component	MtrR, FarR
*glnP*	NGO2012	-10.0	-10.0	ABC-type amino acid transport system, permease component	FarR
*glnQ*	NGO2013	-6.3	-11.1	ABC-type amino acid transporter, ATP-binding protein	MtrR, FarR
					
*apaA*	NGO2014	-6.3	-16.7	Bacterial extracellular solute-binding proteins, family 3	MtrR, FarR
	NGO2016	-3.6	-3.3	Predicted permease	
					
	NGO2096	-6.3	-12.5	SNF family sodium-dependent transporter	
					
*Energy metabolism/Oxidation-Reduction*					
	NGO0108	-4.0	-5.9	NADPH-dependent FMN reductase	Fur, Lrp
*lldD*	NGO0639	-7.1	-11.1	L-lactate dehydrogenase	
*eda*	NGO0713	-3.0	-5.6	KHG-KDPG bifunctional aldolase	
*rpeC*	NGO0758	-4.8	-3.8	Ribulose-phosphate 3-epimerase	
*fdx*	NGO0825	-5.3	-5.3	Ferredoxin	
*cisY*	NGO0918	-3.6	-3.8	Type II citrate synthase	Lrp
*sdhB*	NGO0920	-2.9	-4.2	Succinate dehydrogenase, iron-sulfur protein	Lrp
*sdhA*	NGO0921	-3.7	-3.4	Succinate dehydrogenase, flavoprotein subunit	Lrp
*sdhD*	NGO0922	-2.9	-3.4	Succinate dehydrogenase, membrane anchor protein	
*sdhC*	NGO0923	-1.8	-4.2	Succinate dehydrogenase, cytochrome b556 chain	
*fumC*	NG01029	-2.9	-6.3	Fumarate hydratase	Fur, MtrR
*gpmA*	NGO1258	-4.0	-5.6	Phosphoglyceromutase	Lrp
*nqrA*	NGO1413	-4.0	-7.7	Na(+)-translocating NADH-quinone reductase subunit A	
*nqrB*	NGO1414	-5.0	-8.3	Na(+)-translocating NADH-quinone reductase subunit B	
*nqrC*	NGO1415	-4.2	-8.3	Na(+)-translocating NADH-quinone reductase subunit C	
*nqrD*	NGO1416	-3.8	-16.7	Na(+)-translocating NADH-quinone reductase subunit D	
*nqrE*	NGO1417	-4.3	-12.5	Na(+)-translocating NADH-quinone reductase subunit E	
					
*nqrF*	NGO1418	-3.7	-9.1	Na(+)-translocating NADH-quinone reductase subunit F	MtrR
*dadA*	NGO1808	-3.6	-5.3	D-amino acid dehydrogenase small subunit	
*mqo*	NGO1980	-3.7	-5.0	Malate:quinone oxidoreductase	
					
*Macromolecular biosynthesis*					
*greB*	NGO0262	-3.7	-5.3	Transcription elongation factor	
*parC*	NGO1259	-3.8	-2.9	DNA topoisomerase IV subunit A	
	NGO1261	-6.3	-7.1	S-adenosylmethionine-dependent methyltransferase	
*rplP*	NGO1831.1	-3.2	-6.3	50S ribosomal protein L16	Lrp
*rpsC*	NGO1832	-3.2	-7.7	30S ribosomal protein S3	Lrp
*rplV*	NGO1833	-2.9	-10.0	50S ribosomal protein L22	
*rpsS*	NGO1834	-2.8	-12.5	30S ribosomal protein S19	
*rplD*	NGO1837	-2.2	-7.1	50S ribosomal protein L4	
*rspJ*	NGO1841	-2.7	-7.1	30S ribosomal protein S10	Lrp
*rpoC*	NGO1850	-3.3	-5.0	DNA-directed RNA polymerase subunit beta'	
*rpoB*	NGO1851	-2.1	-4.5	DNA-directed RNA polymerase subunit beta	
					
*Cell Structure*					
*nspA*	NGO0233	-3.1	-3.7	Outer membrane protein (Probable Opa protein)	
GNA2132	NGO1958	-5.3	-8.3	Predicted lipoprotein GNA2132	
*lgtG*	NGO2072	-7.7	-6.3	Probable lipooligosaccharide glycosyl transferase G	
*Adaptation and stress response*					
*cspA*	NGO0410	-3.8	-3.3	Cold shock protein	
*trxB*	NGO0580	-2.6	-4.8	Thioredoxin reductase	NmlR
*trx1*	NGO0652	-5.3	-6.3	Thioredoxin I	Fur
*cstA*	NGO1064	-3.8	-12.5	Putative carbon starvation protein	
*mtrF*	NGO1368	-7.7	-25.0	Antibiotic resistance efflux pump component	MtrR, FarR
*sspB*	NGO2131	-4.5	-3.6	Protease specificity-enhancing factor	
					
*Hypothetical or unassigned*					
	NGO0554	-4.5	-5.0	Hypothetical protein	Fur
	NGO0635	-4.8	-3.6	Hypothetical protein	
	NGO1065	-4.8	-7.7	Hypothetical protein	
	NGO2097	-3.7	-3.1	Conserved hypothetical protein	
					
*Small RNA*					
*nrrF^e^*		-4.8	-7.7	Fur-repressed small regulatory RNA	Fur

*^a ^*Gonococci grown in an anaerobe chamber on plates containing 5 mM nitrite.

*^b ^*The fold change for each replicate was calculated by comparing RPKM values of anaerobically grown gonococci to RPKM values of aerobically grown gonococci. A positive value represents an induction of gene expression anaerobically, and a negative value represents a repression of gene expression anaerobically

*^c ^*Protein definitions are derived from the annotated FA1090 genome (NCBI), and/or the NCBI conserved domain database [33].

*^d ^*(Fur) Genes that have been shown to be directly regulated by Fur in Sebastian *et al*., (2002) [95] and/or Jackson *et al*., (2010) [75]. (RpoH) Genes reported to be upregulated during RpoH overexpression in Gunesekere *et al*., (2006) [23]. (LexA) Genes reported to be in the LexA regulon by Schook *et al*., (2010) [50]. (Lrp) Genes found to be differentially expressed in a *N. meningitidis *Δ*lrp *mutant (NMB0573, closest gonococcal homolog is NGO1407, NCBI) by microarray analysis in Ren *et al*., (2007) [46]. (MtrR) Genes found to be differentially expressed in a gonococcal Δ*mtrR *mutant by microarray analysis in FolsterΔ *et al*., (2009) [56]. (FarR) Genes reported to be in the FarR regulon by Friedrich *et al*., (2007) [54]. (NmlR) Genes reported to be in the NmlR regulon by Kidd *et al*., (2005) [47]. (FNR) Genes found to be differentially expressed microaerobically in a Δ*fnr *mutant with and without nitrite by microarray analysis in Whitehead *et al*., (2007) [7]. (NsrR) Genes reported to be in the NsrR regulon by Isabella *et al*., (2007) [3] and Isabella, (2010) [19].

*^e ^*Fur-repressed small regulatory RNA described by Ducey *et al*., (2009) [96].

As expected, anaerobic expression levels cluster at higher RPKM values in the set of anaerobically induced genes (Figure 1B), while the opposite is true in anaerobically repressed genes (Figure 1C).

### Secondary verification of differentially expressed genes

**Figure 2:**
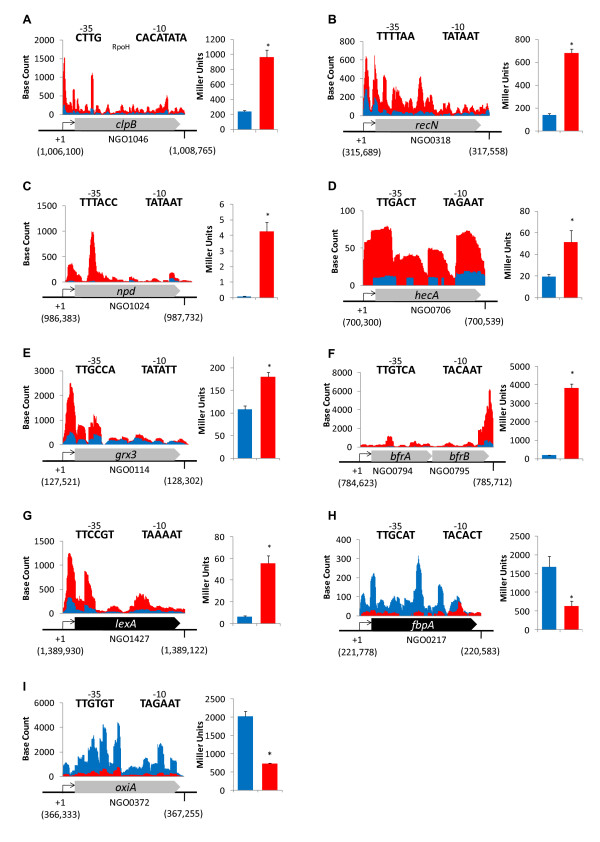
**Secondary verification of RNA-seq results by translational promoter-*lacZ *fusion**. On the right-hand side of each panel, translational *lacZ *fusions confirm that expression of (A) *clpB*, (B) *recN*, (C) *npd*, (D) *hecA*, (E) *grx3*, (F) *brfA*, and (G) *lexA *is anaerobically induced, and that expression of (H) *fbpA *and (I) *oxiA *is anaerobically repressed. For each panel, a prediction of -10 and -35 elements is given above the gene schematic. The predicted transcriptional start site, in parenthesis under the +1, is reported as the chromosomal location according to the annotated FA1090 genome (NCBI). Genes colored in grey are encoded on the positive strand, while genes colored in black are encoded on the negative strand. Above each gene schematic, raw RNA-seq data from .wig files are plotted. The base count is representative of the number of times each base was mapped by a 50 bp RNA sequence read from Replicate 1 (normalized to take into account slight differences in total mapped reads between the two samples). Blue bars represent aerobic base reads and β-galactosidase activity, while red bars represent anaerobic base reads and β-galactosidase activity. Genes are not drawn to the same scale. Results for β-galactosidase activity are presented as the mean + SD of 16 determinations. (*) indicates a p-value less than 0.001.

**Figure 3:**
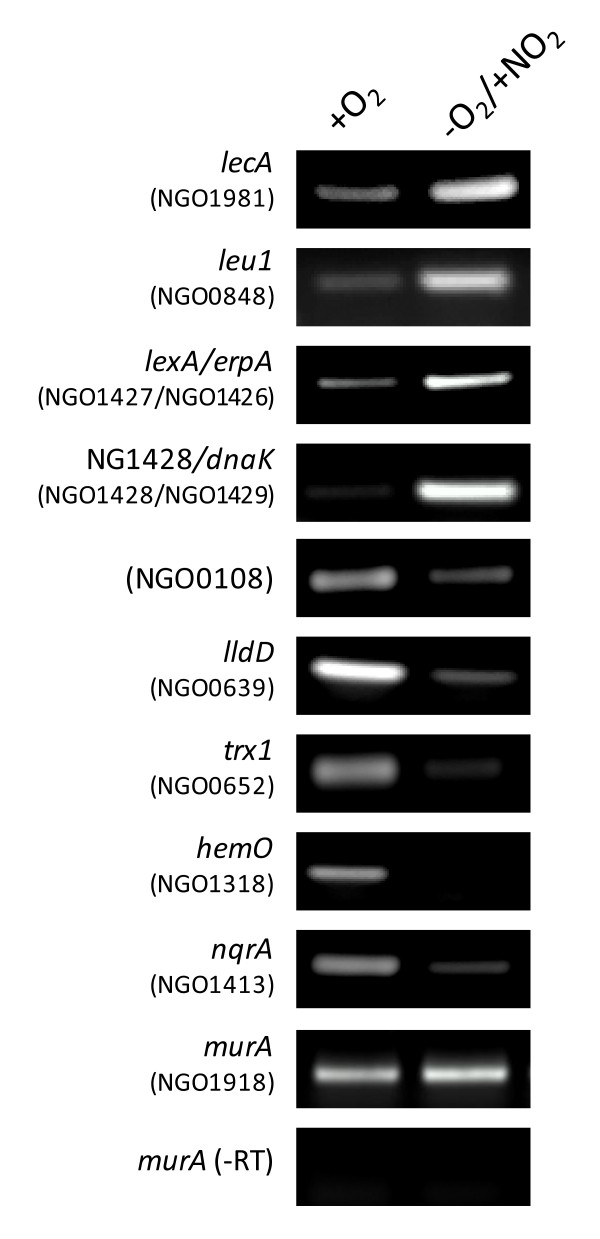
**Secondary verification of RNA-seq results by RT-PCR**. RT-PCR verifies that *lecA *and *leu1 *are anaerobically induced, and that NGO0108, *lldD*, *trx1*, *hemO*, and *nqrA *are anaerobically repressed. RT-PCR of the *murA *transcript (NGO1981), which was shown to have an equal expression level aerobically and anaerobically (See additional file 1: Supplementary Table S1), was used as a loading control. (-RT) signifies that reverse transcriptase was not included in the reaction. For the anaerobically induced genes *lexA*/*erpA *and NG1428/*dnaK*, primers were used that amplified across the 3'end of the *lexA *coding region into the 5' end of the *erpA *coding region, and from the 3' end of the NG1428 coding region into 5'end of the *dnaK *coding region, respectively. Amplification across these intergenic regions suggests that these genes are cotranscribed.

Many of the genes found to have been differentially expressed by RNA-seq have previously been determined to be members of the Neisserial FNR or NsrR regulons, validating the use of this technique to monitor transcriptome changes (Table 1) [3,7,18,19]. To further corroborate the RNA-seq data, several genes shown to be differentially expressed were selected for secondary confirmation. Analysis of translational promoter-*lacZ *fusions or RT-PCR was used to accomplish this task (Figure 2 and 3). Analysis of translational promoter-*lacZ *fusions demonstrated that the genes encoding heat shock protein, ClpB (NGO1046), DNA repair enzyme, RecN (NGO0318), nitropropane dioxygenase, Npd (NGO1024), filamentous hemagglutinin, HecA (NGO0706), glutaredoxin, Grx3 (NGO0114), bacterioferritin, BfrA (NGO0794) and repressor protein LexA (NGO1427) were significantly upregulated anaerobically (Figure 2A-G). RT-PCR confirmed that 2-isopropylmalate synthase, *leu1 *(NGO0848), and adhesin, *lecA *(NGO1981), were also increased in expression anaerobically (Figure 3).

Translational promoter-*lacZ *fusions to the genes encoding the iron-binding protein, FbpA (NGO0217), and the solute binding protein, OxiA (NGO0372) confirmed that these genes are anaerobically repressed (Figure 2 H, I). RT-PCR confirmed that NGO0108, L-lactate dehydrogenase, *lldD *(NGO0639), thioredoxin, *trx1 *(NGO0652), heme oxygenase, *hemO *(NGO1318), and NADH dehydrogenase component, *nqrA *(NGO1413) were also anaerobically repressed (Figure 3).

Analysis of raw RNA-seq data allows for the prediction of transcription start sites, -10, and -35 elements, as has been described in RNA-seq projects in both *Helicobacter pylori *and *Listeria monocytogenes *[20,21]. Compared to *E. coli*, the use of alternative sigma factors in the gonococcus is very limited. Gonococci have no sigma-54 homolog, and only utilize sigma factors in the sigma-70 family. This class of sigma factors recognize -10 and -35 elements within bacterial promoters [22]. The predictions of transcriptional elements for the individual genes selected for secondary analysis are shown in Figure 2. These predictions were generally a high match to the *E. coli *consensus for transcriptional elements (-35, 5'-TTGACA, -10, 5'-TATAAT) or the α-proteobacteria consensus for RpoH-dependent transcriptional elements (-35, 5'-CTTG, -10, 5'-CC/TTATNTNNG) [23]. The ability to predict the location of these transcriptional elements in a large scale manner will prove very useful in the search for potential regulatory sites, and will aid in future work to define gonococcal transcriptional networks.

### Differentially expressed genes involved in macromolecular biosynthesis

**Figure 4:**
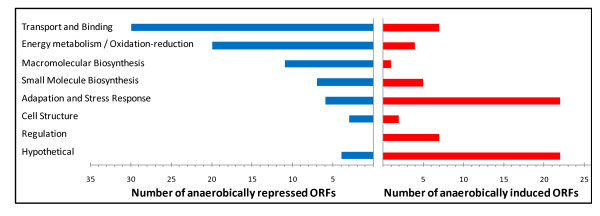
**Functional categories of anaerobically repressed and anaerobically induced genes**. The genes from Table 1 were broadly categorized according to their biological function. Each bar represents the actual number of genes. Blue bars indicate anaerobically repressed genes, and red bars represent anaerobically induced genes.

The genes found to be differentially expressed in response to anaerobic growth were broadly characterized according to their putative function (Table 1, Figure 4). Several genes encoding proteins involved in macromolecular synthesis displayed decreased expression under anaerobic conditions. The synthesis of ribosomal proteins is strongly related to growth rate, and ribosomal protein synthesis has been documented to decrease during times of energy deficiency [24-26]. For facultative anaerobes, growth in an oxygen-limited environment is more energetically-deprived than in aerobic conditions. The reduced synthesis of ribosomal proteins is indicative of adaptation to a slower growth rate following recovery from a nutritional shift-down. The reduced expression of RNA polymerase β an β' subunits is also indicative of this (Table 1), and has been observed in *E. coli *as well [27].

### Genes involved in transport and binding

Many genes encoding proteins involved in transport and binding were found to be anaerobically repressed (Table 1, Figure 4). Most of these proteins are involved in the transport of amino acids, sugars, or other growth metabolites into the cell. This result comes as no surprise, as a slower anaerobic growth rate would result in decreased demand and slower utilization of such substrates. Proteins of this type were also shown to be downregulated anaerobically in *E. coli, P. aeruginosa*, and *S. enterica *[28-30]. Three repressed genes, *fbpA*, *hemO*, and *ompU*, are involved in the acquisition and/or transport of iron into the cell. In *S. enterica*, expression of the gene encoding iron acquisition protein, SitA, was also found to be repressed anaerobically [30].

While genes involved in iron acquisition and transport are repressed anaerobically, the genes encoding the iron-storage bacterioferritin proteins, BfrA and BfrB are induced (Table 1, Figure 2F). Three other proteins implicated in binding that were shown to be anaerobically induced, LecA (Figure 3), HecA (Figure 2D), and fHBP, may be involved directly in the host-pathogen interaction. The role of LecA in gonococci is unclear, but a LecA homolog in another member of the *Neisseriaceae *family, *Eikenella corrodens*, has been shown to be a part of an adhesin complex important in adherence to a variety of host tissues [31]. The gonococcal HecA protein contains a hemagglutination domain that, in other bacteria, has been shown to promote bacterial aggregation or host attachment [32,33]. The role of Factor H binding protein (fHBP) as it relates to gonococcal pathogenesis has not been examined, however, in *N. meningitidis*, fHBP expression on the cell surface was shown to be a means of immune evasion that rendered meningococci resistant to complement-mediated killing [34].

### Genes involved in energy production/oxidation-reduction

Unsurprisingly, numerous genes encoding proteins involved in energy metabolism were found to be anaerobically repressed (Table 1). Gonococci use a combination of the Entner-Douderoff and pentose phosphate pathways in the utilization of glucose [35]. Key enzymes involved in these pathways were found to be anaerobically repressed, as well as several enzymatic components of the Krebs cycle. Anaerobic repression of genes involved in central intermediary metabolism has also been observed in other bacteria, including *E. coli*, *P. aeruginosa*, and *B. subtilus *[28,29,36].

Interestingly, gonococci contain two major NADH dehydrogenases capable of coupling the oxidation of NADH to the reduction of ubiquinone. One of these dehydrogenases is the prototypical H^+^-translocating 14-subunit complex encoded by the *nuo *operon (NGO1737-NGO1751). The second is a seven-subunit Na^+^-translocating complex encoded by the *nqr *operon (NGO1413-NGO1418, NCBI). Though genes in the *nuo *operon did not meet the criteria used in this study to define differential expression, there was an approximate two-fold level of anaerobic repression across its entire length (See additional file 1: Supplementary Figure S1). The *nqr *operon, however, demonstrated a significant decrease in anaerobic expression (Table 1, Figure 3).

Na^+^-translocating NADH dehydrogenases were originally discovered and characterized in marine bacteria, though it has now been revealed that several pathogenic species encode such complexes [37]. Transport of sodium ions into the periplasmic space through the Nqr complex generates an electrochemical membrane potential that plays a role in solute import, ATP synthesis, and, for organisms that are motile, flagellar rotation [37,38]. The flow of electrons through the active portions of the electron transport chain would be expected to decrease anaerobically when: [1] oxygen is unavailable, rendering the terminal cytochrome oxidase (complex IV) inactive, [2] enzymatic components of the Krebs cycle are repressed, resulting in decreased production of reducing equivalents, and [3] growth is dependent on high potential alternative electron acceptors such as NO_2_^- ^and NO. Furthermore, neither the nitrite or nitric oxide reductases are thought to be capable of pumping protons across the membrane [39]. Downregulation of *nqr *would decrease the electrochemical potential across the membrane (ΔΨ), thus decreasing the proton motive force [40]. Because expression of the *nuo *operon is not affected to the same degree anaerobically, this would now result in a greater proportion of NADH oxidation being coupled to ATP production through H^+ ^translocation. Also, many proteins involved in the transport of metabolites into the cell are repressed anaerobically. Downregulation of the Nqr complex may add another level of regulation to cellular transport. It stands to reason that the activity of solute transporters that rely on the sodium motive force would decrease when fewer Na^+ ^ions are pumped across the membrane [38].

The function and regulation of the most highly induced gene involved in anaerobic growth, the nitrite reductase *aniA*, has been thoroughly characterized, and has been shown to be required for anaerobic growth on nitrite [4]. Interestingly, gonococci contain a gene encoding a 2-nitropropane dioxygenase-like protein that was found to be induced anaerobically (NGO1024, Table 1, Figure 2C). This would seem unusual, as gonococci would not be expected to encounter the toxic nitroalkanes utilized by Npd proteins. Furthermore, molecular oxygen is consumed in the Npd reaction mechanism [41]. Further work should determine if this protein plays a novel role in gonococci, possibly microaerobically when oxygen would be present.

Gonococci also showed increased anaerobic expression of a gene encoding electron transfer flavoprotein-ubiquinone oxidoreductase, EftD (NGO1396), a protein containing 57% identity (74% similarity) to human EftD. The presence of *eftD *in bacteria is rare, and its role has only been studied in plants and mammals. In *Arabidopsis thaliana*, EftD was shown to be upregulated in times of carbon starvation, when genes involved in glycolysis and the Krebs cycle were reduced in expression, as is observed when gonococci are grown anaerobically [42]. In *A. thaliana*, it was reported that EftD can act as an electron acceptor from various dehydrogenases involved in amino acid catabolism and fatty acid degradation. The reduction of ubiquinone by EtfD was proposed to be a mechanism of utilizing alternative respiratory substrates to feed into the electron transport chain [42]. EftD upregulation in *N. gonorrhoeae *could indicate a shift to the use of alternative growth substrates under anaerobic conditions.

### Genes involved in small molecule biosynthesis

Anaerobically, several genes involved in the synthesis of amino acids were found to be repressed (Table 1), which has also been observed in other bacteria [28,29]. Genes involved in the synthesis of iron-sulfur clusters were also repressed. This result is not entirely unexpected, as many iron-sulfur cluster containing proteins involved in respiration were shown to be downregulated anaerobically. The demand for iron-sulfur clusters may therefore be reduced under anaerobic conditions. Also, because it is common for the Fe-S clusters in Fe-S cluster-containing proteins to be oxygen-sensitive, a lower rate of oxygen-induced Fe-S cluster turnover would be expected under anaerobic conditions [43].

Unlike other amino acid biosynthetic genes, the gene encoding Leu1, the first committed step in leucine biosynthesis, was found to be induced anaerobically (Table 1, Figure 3). This could be indicative of a decrease in intracellular leucine concentration, as *leu1 *(*leuA*) is repressed by leucine in *E. coli *and other organisms [44]. In *E. coli*, the anaerobic stimulon is intimately associated with global regulation through the leucine responsive regulatory protein, Lrp. [28,45]. In *N. meningitidis*, the reported Lrp regulon demonstrated striking overlap to many of the genes in the gonococcal anaerobic stimulon (Table 1), particularly in regards to proteins involved in glucose utilization, the Krebs cycle, and ribosomal assembly [46]. Future work will examine whether gonococcal Lrp plays a role in control of the anaerobic stimulon.

### Genes involved in transcriptional regulation

The expression of several regulatory proteins was found to be induced under anaerobic conditions, though no regulatory proteins appeared to be repressed (Table 1, Figure 4). The gene encoding ArsR (NGO1562), a regulator involved in *norB *repression in response to iron, was shown to be moderately upregulated [3]. It is unclear if ArsR regulates any other target genes. The FNR-regulated NmlR protein has been shown to act as both an activator and repressor of gene expression, and genes in the NmlR regulon are purported to be involved in resistance to nitrosative stress [47,48]. Though there was an anaerobic increase in *nmlR *expression, none of the genes in the NmlR regulon, except for *nmlR *itself, were found to be upregulated. One member of the regulon, the thioredoxin reductase *trxB *(NGO580), was shown to be significantly downregulated (Table 1). Surprisingly, in a recent study, gonococcal *trxB *mutants were found to be susceptible to killing by NO, though this property was correlated with low expression of *aniA *and *norB *[49]. It stands to reason, therefore, that genes in the NmlR regulon may be important in adaptation to anaerobic conditions or in certain environments where reactive oxygen or nitrogen species are present, but they are possibly not as important once the anaerobic respiratory chain becomes highly induced. In this circumstance, NorB is likely able to reduce NO levels below a threshold needed to induce the activation function of NmlR (NO itself may not be a direct activating signal for NmlR) [47]. Under anaerobic conditions, and in the absence of oxygen or reactive oxygen species (ROS), NO may not need to be detoxified, as toxic NO-derived reactive nitrogen compounds will not be produced. In this instance NO would preferentially be used as an energy source.

The gene encoding LexA was found to be upregulated anaerobically (Figure 2G). This transcriptional regulator was proposed to be important in regulating genes involved in defense against ROS [50]. LexA controls a small regulon in gonococci that includes itself, DNA repair protein, *recN*, and a gene of unknown function, NGO1428. LexA has been demonstrated to sense hydrogen peroxide through thiol modification of a single cysteine residue [50], though the anaerobic induction of the LexA regulon observed here suggests that LexA may also sense NO. Interestingly, analysis of the raw RNA-seq data and RT-PCR showed that under conditions where the LexA regulon was induced, significant transcriptional read-through occurred beyond *lexA *into the adjacent *erpA *gene (NGO1426), as well as past the divergently transcribed NGO1428 gene into the adjacent *dnaK *gene (NGO1429, Figure 3). Both *erpA *and *dnaK *are members of the RpoH regulon [23], suggesting that there may be crosstalk between the RpoH and LexA regulons.

Though transcriptional read-through may account for part of the anaerobic induction of *erpA *and *dnaK*, our data suggest that RpoH activity itself was likely modulated anaerobically. Although there was no significant increase in the quantity of RpoH transcripts at the 24 hr time point at which the cells were harvested, control of *rpoH *at the transcriptional level plays a relatively minor role in maintaining RpoH levels in the cell [51]. A majority of RpoH regulation is posttranscriptional. Translation of RpoH is inhibited by secondary structure in the *rpoH *mRNA, and activity of RpoH can be further modulated by the chaperone DnaK [23]. Including *erpA *and *dnaK*, eight out of the twelve genes previously demonstrated to encompass the gonococcal RpoH regulon were found to be significantly upregulated anaerobically, including genes encoding a transcriptional regulator of unknown function, MarR (NGO1244), dihydrofolate reductase, FolA (NGO1694), putative DNA-binding protein, CreA (NGO0570), and chaperone proteins ClpB (NGO1046), GrpE (NGO1422), and DnaJ (NGO1901, Table 1, Figure 2A) [23]. The anaerobic induction of genes in the RpoH regulon may be of significance *in vivo*, as these genes have been shown to contribute to epithelial cell invasion [52].

The gene encoding FarR (NGO0058) was found to be anaerobically induced (Table 1). FarR was originally described for its role in repression of the *farAB *operon, which is involved in fatty acid resistance [53]. Analysis of the RNA-seq data demonstrated that *farAB *expression was not altered anaerobically, suggesting that this operon may already be fully repressed under standard laboratory conditions. A recent study established that FarR, possibly indirectly, was also responsible for the repression of two ABC transporter cassettes (NGO372-NGO0374 and NGO2011-NGO2014) as well as the gene encoding the multiple transferable resistance protein, MtrF (NGO1368) [54]. All of these genes were found to be anaerobically repressed by RNA-seq analysis (Table 1, Figure 2I), suggesting that FarR is yet another player in the anaerobic lifestyle of *N. gonorrhoeae*. Interestingly, expression of FarR itself was determined to be controlled by repression through MtrR, a regulator of the MtrCDE efflux pump involved in resistance to antimicrobial agents [55]. MtrR was recently shown to act in a global fashion, regulating many genes, including RpoH [56]. Many of the genes in the reported MtrR regulon also appear to be part of the anaerobic stimulon in gonococci, suggesting a potential role for this regulator in anaerobic growth as well [56] (Table 1).

### Genes involved in adaptation and stress response

In addition to the RpoH regulon, many genes involved in response and adaptation to stress were found to be anaerobically induced. Several genes encoding proteins involved in DNA repair were found to be upregulated anaerobically (Table 1). RecN is involved in recombinational repair of DNA damage, and has been shown to protect gonococci from both oxidative and nonoxidative mechanisms of killing [57,58]. RecX (NGO1053) has been demonstrated to be involved in enhancement of RecA-mediated processes, including repair of DNA damage [59]. A gene of unknown function, NGO1022, was also upregulated anaerobically, and encodes a DEDDh-family protein with a high level of homology to DnaQ (NGO0973), the epsilon subunit of DNA polymerase III [33]. As the epsilon subunit of DNA polymerase III contains 3' → 5' exonuclease activity and is involved in DNA mismatch repair, this DnaQ-like protein may also function in the repair of DNA damage [60].

Several genes typically involved in resistance to oxidative stress were found to be induced anaerobically, including those encoding glutaredoxin, Grx3, cytochrome C peroxidase, CCP (NGO1769), tellurite resistance protein, TehB (NGO1161), and nitroreductase-like protein, NtrA (NGO1702, Table 1, Figure 2E). CCP was shown to protect gonococci from hydrogen peroxide mediated damage, but not to be involved in protection from reactive nitrogen species [61,62]. Activation of CCP by FNR suggests that gonococci may typically be exposed to ROS under anaerobic or microaerobic conditions *in vivo*. Proteins similar to TehB were originally described for their role in resistance to tellurite, however, gonococci do not inhabit an environment where tellurite would be encountered. In *Haemophilus influenzae*, it was recently shown that a deletion of *tehB *resulted in increased sensitivity to oxidizing agents including hydrogen peroxide, suggesting a dual role for this protein [63]. The protein encoded by NGO1702 was shown to contain a nitroreductase-like domain [33], and to contain 54% identity (66% similarity) to a novel nitroreductase in *Staphylococcus aureus*, NtrA. In *S. aureus*, NtrA was shown to contribute to nitrosative stress resistance through its S-nitrosoglutathione (GSNO) reductase activity [64]. Two other proteins, DnrN and ErpA, were also demonstrated to be upregulated anaerobically. DnrN has been implicated in the repair of iron sulfur clusters damaged by oxidative or nitrosative stress [65], and ErpA may be important in maintaining the iron-sulfur cluster status of specific proteins in cells growing under stressful conditions [66].

Interestingly, genes encoding two Nudix-family hydrolases, NudH (NGO1334) and NGO1566, as well as a histidine triad family protein, Hit (NGO1273), were all found to be upregulated anaerobically (Table 1). Analysis of the conserved domains of these open reading frames reveals the greatest level of homology to proteins involved in metabolism/hydrolysis of diadenosine tetraphosphate (Ap_4_A) [33]. Ap_4_A is an important and ubiquitous signaling molecule in nature, and has been implicated in the maintenance and regulation of vital cellular functions [67,68]. As a bacterial second messenger, Ap_4_A has been shown to be involved in modulating chaperone and heat shock protein activity, coupling DNA replication to cell division, and altering the ability of cells to use alternate carbohydrate sources, though these may be just a few of its functions [68,69]. If allowed to accumulate unchecked, high Ap_4_A concentration can interfere with a number of ATP-dependent reactions [70]. In *E. coli*, *Salmonella typhimurium*, and *Bartonella bacilliformis*, deletion of the *nudH *gene resulted in gross deficiencies in the ability of these organisms to invade mammalian cells [68,69]. The role of Ap_4_A as a second messenger in gonococci has yet to be evaluated thus far, though it appears that the metabolism of this molecule may be modulated anaerobically, making it an interesting target for future study.

### Hypothetical proteins regulated anaerobically

Many genes encoding hypothetical proteins were found to be induced anaerobically (Table 1, Figure 4). One gene, listed as DUF331 in Table 1, was not found in the FA1090 genome annotation, rather it was discovered in proximity to the iron sulfur cluster assembly operon (*isc*) using raw RNA-seq data. Using this data, it was discovered that the *isc *operon spanned the *iscR *gene (NGO0637) through *hesB *(NGO0632), making it longer than the current annotation would suggest. NGO0634 probably does not encode a protein, and no reads were mapped on the positive strand at this location (See additional file 1: Supplementary Table S1). A high level of transcription was found at an unannotated putative open reading frame with an ATG start codon on the positive strand at chromosomal coordinate 621,869 (NCBI). There was a high match to consensus -35, -10, and RBS sequences upstream of this open reading frame, and transcription of this gene partially overlapped the 3' end of the *isc *operon. This anaerobically induced gene encodes a protein with a conserved domain of unknown function (DUF331). This is just an example of how RNA-seq data can be used to locate and correct annotation errors in the gonococcal genome. The large quantity of hypothetical proteins that are differentially expressed in response to anaerobiosis may make appealing targets for future study. Many of these annotated proteins are small and Neisseria-specific. Future work should determine if these are akin to small proteins in *E. coli *that have been shown to accumulate in response to stress [71]. Alternatively, these small open reading frames could be mis-annotations, and may be involved in transcription of regulatory RNA. Current work involves integrating raw RNA-seq data in a genome-wide search for regulatory RNA.

### *N. gonorrhoeae *encodes an anaerobically induced sRNA

In *E. coli *and several other Enterobacterial species, recent studies have described the anaerobic induction and function of a small regulatory RNA termed *fnrS *[72,73]. In *E. coli*, the regulation of this sRNA was determined to be relatively complex. Maximal *fnrS *expression occurred anaerobically, however, the available carbon source and terminal electron acceptor present also impacted levels to a lesser extent. Anaerobic induction of *fnrS *was shown to be FNR-dependent and mediated by FNR binding to a class-II activation site centered at -41.5 with respect to the transcription start site [72]. No *fnrS *homolog has been observed in bacteria outside of the *Enterobacteriaceae *family, however, previous microarray analysis of the gonococcal FNR regulon identified a small FNR-activated transcript of unknown function. Coincidently, this transcript was also determined to be the most highly induced by FNR [7].

**Figure 5:**
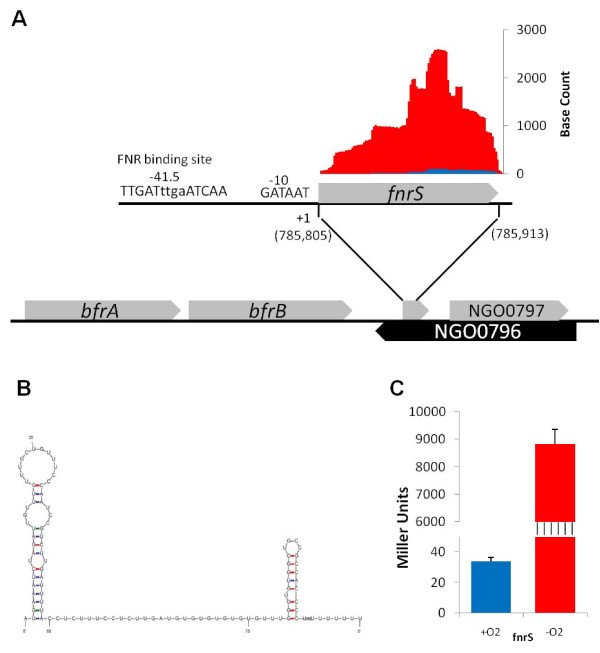
***N. gonorrhoeae *encodes an anaerobically induced small RNA**. (A) A schematic representation of the location of the *fnrS *coding region is given, with genes colored in grey encoded on the positive strand, and genes colored in black encoded on the negative strand (according to the NCBI annotation). Above the *fnrS *gene schematic, raw RNA-seq data from .wig files are plotted. The base count is representative of the number of times each base in the transcript was mapped by a 50 bp RNA sequence read from Replicate 1 (normalized to take into account slight differences in total mapped reads between the two samples). Blue bars represent aerobic base reads and red bars represent anaerobic base reads. A prediction of the -10 element and class-II FNR binding site is given to the left of the *fnrS *gene. The chromosomal location of the predicted transcriptional start site, in parenthesis under the +1, is reported. (B) The lowest energy structure of the *fnrS *transcript as predicted by the *Mfold *program http://mfold.rna.albany.edu/ is displayed. (C) The β-galactosidase activity of an *fnrS::lacZ *transcriptional fusion is presented as the mean + SD of 16 determinations (p < 0.001).

This small FNR-induced transcript is located within the coding region and on the opposite strand of NGO0796, flanked on the 5' end by the bacterioferritin genes, *brfA *and *bfrB*, and on the 3' end by an Xre-family repressor (NGO0797) end by an (Figure 5A). Though not included the FA1090 genome annotation, analysis of the raw RNA-seq data confirms that this transcript is highly induced anaerobically (Table 1, Figure 5A). The region upstream of this transcript also contains a perfect match to the *E. coli *FNR consensus sequence (5'-TTGATnnnnATCAA) located at -41.5 with respect to the transcription start site. Secondary structure prediction of this 108 nt transcript was performed by the *Mfold *program http://mfold.rna.albany.edu/, and the most stable predicted structure is shown in Figure 5B. This predicted structure is typical of a small regulatory RNA, with a 5' stem loop followed by an unstructured region and a 3' Rho-independent terminator (Terminator prediction performed at: http://transterm.cbcb.umd.edu).

To confirm that this small RNA is expressed and induced anaerobically, a transcriptional fusion to *lacZ *was constructed. In this construct, the promoter region from the induced transcript (from -120 to -1) was cloned in front of the leader region of *lacZ*, which provided spacing and an RBS for efficient translation. Aerobic β-galactosidase activity from this transcriptional fusion was low, but was induced 260-fold anaerobically (Figure 5C), validating the RNA-seq data and the work of Whitehead, *et al*. (2007) [19]. To show that transcription was halted at the predicted Rho-independent terminator, a transcriptional fusion was generated in which the entire small RNA, including the promoter region (from -120 to +111), was fused to the leader region of *lacZ*. No β-galactosidase activity was observed from this fusion gene aerobically or anaerobically, confirming the terminator prediction and the raw RNA-seq data (data not shown, Figure 5). The first three nucleotides of this small transcript are AUG. To ensure that this transcript is indeed a small RNA and not a leaderless protein-coding message, a translational fusion was constructed. A leaderless *lacZ *gene was fused in-frame past nucleotide +9 in the transcript. No β-galactosidase activity was observed in this translational fusion under aerobic or anaerobic conditions (data not shown). For these reasons, we feel that this small anaerobically induced transcript should be termed *fnrS*. Much work needs to be completed to define the role, if any, of this gonococcal FnrS transcript in gene regulation, however, homologs of genes in the *E. coli *FnrS regulon that were found to be anaerobically repressed in gonococci, including *gpmA *(phosphoglycerol mutase) and *mqo *(malate:quinone oxidoreductase), will make interesting candidates to study for potential interactions [72]. Future work will employ a similar RNA-seq approach to define the prospective FnrS regulon in gonococci.

### Overlap of the anaerobic, iron, and hydrogen peroxide stimulons

**Figure 6:**
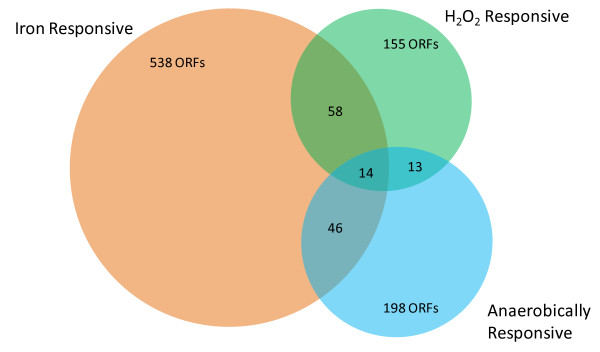
**Overlap of the anaerobic, iron, and hydrogen peroxide responsive stimulons in *N. gonorrhoeae***. Genes found to be differentially expressed in response to anaerobiosis, iron, and hydrogen peroxide were compared in order to discover anaerobically regulated genes that were responsive to additional environmental signals. A Venn diagram was constructed to display the extent of overlap between these stimulons. Data for the iron responsive stimulon was derived from Jackson, *et al*., (2010) [75]. Data for the hydrogen peroxide responsive stimulon was derived from Stohl, *et al*., (2005) [74].

*In vivo*, *N. gonorrhoeae *will be faced with a multitude of environmental stresses, including oxygen and iron limitation, as well as exposure to reactive oxygen species. Previous studies have employed genome-wide approaches to define the gonococcal iron and hydrogen peroxide responsive stimulons [74,75]. Genes differentially expressed in response to these stimuli were compared to the anaerobic stimulon to search for similarities in gene expression between these environmental signals (Figure 6, Table 2).

**Table 2:** Comparison of the anaerobic stimulon with the iron and hydrogen peroxide responsive stimulon

Gene	ORF designation				Definition
*Common elements of the anaerobic and iron responsive stimulon*					
					
		-O_2_/+O_2_a	-Fe/+Fe^b^		
*farR*	NGO0058	3.9	-2.0		MarR family transcriptional regulator
*gluP*	NGO0142	-3.7	2.9		Glucose/galactose transporter
*nhaC*	NGO0143	-3.1	-2.3		Na+/H+ antiporter (NhaC)
*amtB*	NGO0198	-4.3	-14.2		Ammonium transporter AmtB
*nspA*	NGO0233	-3.4	2.6		Outer membrane protein
*greB*	NGO0262	-4.5	-1.8		Transcription elongation factor
	NGO0373	-7.4	-2.5		Amino acid ABC transporter, permease protein
	NGO0377	-7.4	-150.4		Probable transmembrane transport protein
*cspA*	NGO0410	-3.6	3.2		Cold shock protein A
	NGO0492	7.7	1.8		Putative phage associated protein
	NGO0506	13.6	-3.2		Putative phage associated protein
	NGO0635	-4.2	4.2		Hypothetical protein
*lldD*	NGO0639	-9.1	3.9		L-lactate dehydrogenase
*pdxH*	NGO0658	4.9	1.8		Pyridoxamine 5-phosphate oxidase
*eda*	NGO0713	-4.3	6.9		KHG-KDPG bifunctional aldolase
	NGO0732	2.8	2.7		Neisseria-specific protein
*mobA*	NGO0754	4.1	3.2		Molybdopterin-guanine dinucleotide biosynthesis protein
	NGO0757	3.4	2.2		Putative periplasmic Cpx-family sensor kinase
*bfrA*	NGO0794	12.0	-2.8		Bacterioferritin A
*bfrB*	NGO0795	9.9	-1.9		Bacterioferritin B
*hslR*	NGO0822	3.9	2.4		Heat shock protein (Hsp15)
	NGO0895	5.5	1.8		Neisseria-specific protein, uncharacterized
*sdhA*	NGO0921	-3.6	-2.3		Succinate dehydrogenase flavoprotein subunit
*sdhC*	NGO0923	-3.0	-2.3		Succinate dehydrogenase, cytochrome b556 chain
	NGO0995	3.2	1.9		Neisseria-specific protein, uncharacterized
*hsp33*	NGO1189	5.7	-2.9		Heat shock protein Hsp33 chaperonin
	NGO1261	-6.7	3.4		S-adenosylmethionine-dependent methyltransferase
*lldP*	NGO1361	-8.7	-3.2		L-lactate permease
*nqrB*	NGO1414	-6.7	-2.7		Sodium-translocating NADH-ubiquinone reductase subunit B
*nqrC*	NGO1415	-6.3	-2.1		Sodium-translocating NADH-ubiquinone reductase subunit C
*nqrE*	NGO1417	-8.4	-5.5		Sodium-translocating NADH-ubiquinone reductase subunit E
*nqrF*	NGO1418	-6.4	-3.1		Sodium-translocating NADH-ubiquinone reductase subunit F
*putP*	NGO1552	-8.3	-5.3		Sodium/proline symporter PutP
*ompA*	NGO1559	3.6	2.5		Probable outer membrane protein
*arsR*	NGO1562	4.2	-2.3		ArsR family transcriptional regulator
	NGO1628	6.3	2.1		Conserved hypothetical protein
	NGO1633	82.1	2.1		Putative phage associated protein
	NGO1688	-3.1	3.7		Conserved hypothetical protein
*folA*	NGO1694	3.3	-2.0		Dihydrofolate reductase (FolA)
	NGO1807	-3.3	-3.0		Amino-acid transporter
*dadA*	NGO1808	-4.4	-2.9		D-amino acid dehydrogenase small subunit (DadA)
*yhhF*	NGO1860	3.9	2.2		Probable DNA methylase Symport protein (possible sodium/dicarboxylate
	NGO1957	-7.8	-2.0		symporter)
	NGO2023	4.9	1.9		Conserved hypothetical protein
*sspB*	NGO2131	-4.1	3.3		Stringent starvation protein B
	*nrrF*	-6.2	170†		Fur regulated small regulatory RNA
					
*Common elements of the anaerobic and hydrogen peroxide responsive stimulon*					
					
		-O_2_/+O_2_	+HP/-HP^c^		
*recN*	NGO0318	5.1	3.0		DNA repair protein RecN (recombination protein N)
*dnaB*	NGO0485	5.2	6.5		Replicative DNA helicase
*nmlR*	NGO0602	7.4	-4.8		Transcriptional regulator MerR-family
*nifU*	NGO0633	-5.0	4.6		Fe-S scaffold protein
*iscS*	NGO0636	-5.2	4.1		Cysteine desulferase
*leu1*	NGO0848	5.6	3.3		2-isopropylmalate synthase
*aniA*	NGO1276	28.2	-3.6		Copper-containing dissimilatory nitrite reductase
*grpE*	NGO1422	3.2	5.8		Heat shock protein (HSP-70 cofactor)
*lexA*	NGO1427	4.8	6.3		Transcriptional regulator, repressor
	NGO1428	11.4	3.9		Neisseria-specific protein, uncharacterized
*dnaK*	NGO1429	3.1	10.8		Heat shock protein (HSP-70 chaperone)
*ccp*	NGO1769	8.9	2.7		Probable cytochrome c peroxidase
*dnaJ*	NGO1901	4.0	3.0		Heat shock protein HSP-40/chaperone DnaJ
					
*Common elements of the anaerobic, iron, and hydrogen peroxide responsive stimulon*					
					
		-O_2_/+O_2_	-Fe/+Fe	+HP/-HP	
	NGO0108	-4.9	4.7	6.8	Conserved hypothetical protein (possible oxidoreductase)
*glr3*	NGO0114	5.0	4.7	4.8	Glutaredoxin 3
*fbpA*	NGO0217	-5.2	8.7	5.5	Periplasmic iron-binding protein
	NGO0554	-4.8	24.5	70.6	Hypothetical protein
*trx1*	NGO0652	-5.8	3.2	13.5	Thioredoxin I
*fumC*	NGO1029	-4.6	6.4	8.4	Fumarate hydratase (fumarase C)
*clpB*	NGO1046	7.2	4.3	23.8	Endopeptidase ClpB (heat shock protein)
*pgm*	NGO1258	-4.8	2.0	2.8	Phosphoglycerate mutase
*hemO*	NGO1318	-9.8	8.4	9.8	Heme oxygenase/iron starvation protein
*exbD*	NGO1377	-3.5	3.9	4.4	Transport protein (ExbD)
*exbB*	NGO1378	-4.1	6.5	4.7	Transport protein (ExbB) Sodium-translocating NADH-ubiquinone reductase
*nqrA*	NGO1413	-5.8	-2.3	-2.6	subunit A
					Sodium-translocating NADH-ubiquinone reductase
*nqrD*	NGO1416	-10.3	-4.0	-2.8	subunit D
*lecA*	NGO1981	4.5	2.5	2.5	Adhesin protein

*^a ^*average of RPKM ratios from replicates in Table 1.

*^b ^*Data from Jackson *et al*., (2010) [75], taken from time point where iron depletion had the biggest effect on gene expression. A positive value represents an induction of gene expression under iron depleted conditions, and a negative value represents a repression of gene expression under iron depleted conditions.

*^c ^*Hydrogen peroxide (HP); Data from Stohl *et al*., (2005) [74]. A positive value represents induction of gene expression in the presence of hydrogen peroxide, and a negative value represents a repression in gene expression in the presence of hydrogen peroxide.

† Data from Ducey, *et al*., (2009) [96].

It should be noted that there are caveats in directly comparing these data sets due to the differences in experimental design that exist between each study. The use of microarrays in the iron and hydrogen peroxide studies may be less sensitive than the use of transcriptome sequencing, and therefore it is possible that some additional iron- or hydrogen peroxide-regulated genes may have been missed [16]. Differences in the cutoff level used to define differential expression, the growth medium utilized, and the growth phase of the cells used for RNA isolation between these studies may also impact the results. Though the iron and hydrogen peroxide studies utilized RNA from broth-grown cells in mid-log phase, the growth phase of anaerobically grown gonococcal cells is difficult to determine due to the requirement for growth on solid media, and consequently, this may also affect the comparison of these results with other studies [74,75]. With these factors in mind, a significant overlap does exist between the members of these stimulons, and it should be noted that of the common members, the response is not always predictable. That is, genes regulated in response to these stimuli do not follow a common expression pattern between gene sets, underscoring the complexity of gonococcal gene regulation (Table 2). For example, the *nqr *operon and *bfrA *are both members of the iron stimulon and are both repressed under iron-depleted conditions, however, *nqr *and *bfrA *are repressed and induced, respectively, during anaerobic growth. From this comparison we hope to discover Neisserial genes that may play an important role in survival under a broad range of environmental stress. Analysis of this gene set may also help future work to define transcriptional networks at the regulator level.

### Gonococcal prophage genes are highly induced anaerobically

Somewhat unexpectedly, the RNA-seq data showed that a large subset of the anaerobically induced genes were bacteriophage in origin (47 genes, Table 1). The dsDNA filamentous prophage, NgoΦ1, is believed to contain all of the coding regions necessary to produce functionally active phage particles. In a previous study, phage DNA from NgoΦ1, as well as phage particles that were likely NgoΦ1 -derived, were observed in gonococci, though plaques were not observed [76]. In this study, RNA-seq data showed dramatic anaerobic induction of the NgoΦ1 coding region.

Though no plaques have been observed in anaerobically grown gonococci, this is not the first report of anaerobic prophage induction. In anaerobically grown *Pseudomonas aeruginosa*, prophage genes were shown to be among the most highly induced, and it was suggested that, more specifically, nitric oxide was likely involved in this apparent induction [29]. Evidence has also been provided to show that these anaerobically induced phage are involved in biofilm development and differentiation in *P. aeruginosa *[77]. Current work proposes that gonococcal biofilms are important in pathogenesis. It was recently shown that anaerobic metabolism occurs in the substratum of gonococcal biofilms, and that NO plays an important role in biofilm maintenance [78]. It is interesting to speculate that in the gonococcus, NgoΦ1 may play a role similar to that of anaerobically induced phage in *P. aeruginosa*.

NgoΦ3, NgoΦ4, and NgoΦ5 encode incomplete phage genomes and are probably derived from NgoΦ1 or NgoΦ2 [76]. NgoΦ3, NgoΦ4, and NgoΦ5 were all shown to contain coding regions that were upregulated anaerobically (Table 1). The functions of these genes are unknown, but the presence of prophage sequences in bacterial genomes could have a profound effect on host cell fitness or pathogenicity. A diverse group of bacterial virulence factors, including toxins, have been found to be encoded by genes of phage origin. Some prophage can also contain regulatory proteins that affect expression of genes not encoded by phage [Reviewed in, [79]]. It is certainly possible that none of the anaerobically upregulated gonococcal prophage genes are involved in making active phage particles; alternatively, these genes may have acquired a novel function. Regardless, further research will be required to determine the role of these genes as they relate to anaerobiosis.

### Expression from the cryptic plasmid is induced anaerobically

**Figure 7:**
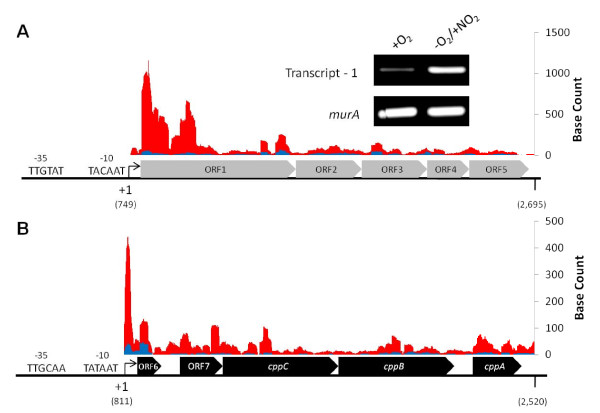
**Expression from the gonococcal cryptic plasmid is induced anaerobically**. Schematic representations of (A) transcript-1 and (B) transcript-2 of the cryptic plasmid are displayed. Genes colored in grey are encoded on the positive strand, while genes colored in black are encoded on the negative strand. The predicted transcriptional start site, in parenthesis under the +1 for each transcript, is reported as the location on the plasmid according to the annotation of Korch *et al*., (1985) [80]. Above each gene schematic, raw RNA-seq data from .wig files are plotted. The base count is representative of the number of times each base was mapped by a 50 bp RNA sequence read from Replicate 1 (normalized to take into account slight differences in total mapped reads between the two samples). Blue bars represent aerobic base reads and red bars represent anaerobic base reads. A prediction of -10 and -35 elements is given to the left of each schematic. RT-PCR confirms that transcript-1 is induced anaerobically.

The gonococcal cryptic plasmid, pJD1, is 4,207 bp long and is found in approximately 96% of gonococcal strains. Despite its predominance, the function and replication mechanism of this plasmid remains largely unknown [80,81]. This plasmid consists of two divergent transcripts, each consisting of five open reading frames (Table 3, Figure 7). Surprisingly, pJD1 transcripts were greatly increased in prevalence anaerobically (Table 3, Figure 7). It appears as though the increase in transcript quantity was not due to an increase in anaerobic copy number, as there was no significant difference in the quantity of plasmid recovered from equal numbers of aerobically or anaerobically grown cells (data not shown). This result implies that the two divergent cryptic plasmid promoters are subject to transcriptional regulation.

**Table 3:** Genes of the cryptic plasmid found to be differentially expressed in response to anaerobiosis.

Gene	**Replicate 1**^***b***^	Replicate 2	**Definition**^***a***^
*Transcript 1*			
ORF1	12.8	9.7	Putative plasmid replicase, RepA
ORF2	4.5	3.1	Hypothetical protein
ORF3	4.5	3.4	Hypothetical protein
ORF4	4.5	2.6	Putative plasmid antitoxin, VapX
ORF5	5.1	5.5	Putative plasmid toxin, VapD
			
*Transcript 2*			
ORF6	18.2	6.0	Hypothetical protein
ORF7	13.3	34.4	Hypothetical protein
*cppC*	19.9	41.6	Putative plasmid mobilization protein, MobA
*cppB*	6.2	7.9	Putative plasmid mobilization protein, MobB, partial only
*cppA*	7.6	8.2	Cryptic plasmid protein A

*^a ^*Protein definitions are derived from Korch *et al*., (1985) [80] or the NCBI conserved domain database [33].

*^b ^*The fold change for each replicate was calculated by comparing total number of reads for each gene in anaerobically grown gonococci to number of reads for each gene in aerobically grown gonococci. Values were normalized to total number of chromosomal reads. A positive value represents an induction of gene expression anaerobically, and a negative value represents a repression of gene expression anaerobically.

In other plasmids, the essential RepA protein has been shown to be involved in regulating gene expression as well as the initiation of plasmid replication (RepA encoded by ORF1 in pJD1, Table 3) [82,83]. In several organisms, DnaK has been shown to facilitate the dissociation of RepA dimers into monomers, resulting in derepression [84,85]. Therefore it is possible that the anaerobic increase in DnaK concentration could result in a decrease in RepA-mediated autorepression (Table 1). Unfortunately, little is known about the replication cycle of pJD1 or the mechanism of RepA binding in gonococci, so this potential regulatory mechanism remains only speculative. An alternate possibility is that an as-of-yet unidentified chromosomally expressed transcription factor or factors acts at the divergent plasmid promoters to regulate their expression.

The effect of increased cryptic plasmid expression on gonococcal physiology is unknown. Previous studies involving pJD1 have provided evidence that ORF1, ORF2, ORF4, ORF5, *cppC*, *cppB*, and *cppA *are capable of forming protein products, and several of these protein products have no homology to any other proteins in the NCBI conserved domain database (Table 3) [33,80,81]. It is uncertain if ORF3, ORF6, or ORF7 are translated. The high frequency and sequence conservation of this plasmid among gonococcal isolates suggests that the plasmid may be important in pathogenesis. The fact that transcription from this plasmid is induced anaerobically is perhaps suggestive that the gene products of this plasmid are important in an anaerobic niche.

In transcript-1, ORF4 and ORF5 encode a putative toxin/antitoxin system analogous to the VapDX systems of *Haemophilus influenzae *and several species of *Actinobacillus *[86,87]. It is assumed that the VapDX system is involved in stable maintenance of pJD1 [88]. Interestingly, in *H. influenza*e, the VapDX locus is located on the chromosome, and VapD has been characterized as a virulence factor. It has been proposed that the ability of VapD to cause an arrest of bacterial translation was responsible for an observed enhancement of bacterial survival within human cells compared to survival in a Δ*vapD *mutant [86]. VapD was postulated to facilitate persistent or chronic mucosal infections under stressful conditions. The role of VapD as it pertains to gonococcal virulence, if any, is unknown, though it may make an attractive target for future study.

## Conclusions

The goal of this study was to define the gonococcal transcriptome in response to anaerobiosis. Unlike previous studies that have looked only at the contributions of individual transcriptional regulators, we used a powerful and sensitive methodology, RNA-seq [16], to look at the environmental condition of anaerobiosis as a whole. By using this method, we were able to show that the anaerobic stimulon in *N. gonorrhoeae *was larger than the FNR and NsrR regulons indentified in previous studies [7,19]. The induction of prophage and cryptic plasmid genes was previously unknown to occur during anaerobiosis. Many new targets for future study have been uncovered, and we hope that other investigators will take an interest in working on these potential projects. Because anaerobic growth is assumed to be a physiologically significant state *in vivo *[4], the results of this study may help to elucidate factors or mechanisms of virulence that have previously been overlooked.

## Methods

### Growth of gonococcal strains

All gonococcal strains were derived from strain F62 (See additional file 2: Supplementary Table S2) and were grown on Difco™GC medium base (Becton, Dickinson and Co., Sparks, MD) plates with 1% Kellogg's supplement (GCK) [89]. A 5 mM concentration of nitrite was added to plates to be used for anaerobic cultures. For RNA isolation and β-galactosidase assays, overnight plate-grown cell cultures were suspended in GCP broth (proteose peptone #3 (15 g), soluble starch (1 g), KH_2_PO_4 _(4 g), K_2_HPO_4 _(1 g), NaCl (5 g)/L dH_2_O) to an OD_600 _of 1.0 and serially diluted tenfold to 10^-4^. A 100 μL volume of the 10^-4 ^dilution was plated onto GCK plates with or without nitrite. Aerobic plate cultures were grown for 14-16 h in a 37°C incubator supplying 5% CO_2_. Anaerobic cultures were incubated in a Coy anaerobic chamber (Coy Laboratory Products, Grass Lake, MI) at 37°C for 20-24 h in an atmosphere of 85% N_2_, 5% H_2_, and 5% CO_2_. Cells were harvested from aerobically and anaerobically incubated plates when colonies were approximately the same size.

### RNA isolation and RT-PCR

Plate-harvested cells were incubated shaking in tubes with 0.1 mm diameter silica beads in RNA*pro*™solution, and RNA was extracted according to manufacturer's instructions (MP Biomedicals, CA). RNA was treated with DNase I (Invitrogen, instructions CA), and integrity was confirmed with a Bioanalyzer (Agilent). RT-PCR was performed with SuperScript^® ^III One-step RT-PCR reagents (Invitrogen, CA). A 125 ng quantity of total RNA was used in each RT reaction, and primers were added at a concentration of 10 μM. Reactions were run as described: 20 min incubation at 45°C to complete the RT reaction, followed by 2 min incubation at 94°C, 23 to 26 subsequent cycles of 15 s at 94°C, 30 s at 55°C, and 30 s at 68°C, and a final incubation at 68°C for 5 min. Samples were electrophoresed on 1% agarose gels for visualization. All RT-PCR experiments were repeated a minimum of two times. Primers available upon request.

### RNA preparation and SOLiD™ RNA-seq

For RNA to be used in RNA-seq, two rounds of mRNA enrichment were performed on total bacterial RNA using MICROBExpress Oligo MagBeads (Ambion, TX). The University of Rochester core facility performed the SOLiD sequencing. The SOLiD™ 3 Plus system was used to read short (50 nt) RNA sequences, and all RNA processing procedures were performed using a SOLiD™ Total RNA-seq Kit as recommended by Applied Biosystems (SOLiD™ Total RNA-seq Kit Protocol, Applied Biosystems, CA). Briefly, RNA was fragmented with RNase III, directionally hybridized and ligated with flanking adapters containing sequences for priming PCR amplification and sequencing reactions. After reverse transcription of the ligated RNA, the cDNA molecules were size selected by PAGE, amplified by PCR (limiting the number of cycles to minimize PCR bias), quantified by real-time PCR, and diluted to a concentration optimal for monoclonal amplification by emulsion PCR, during which copies of template molecules were attached to beads. Beads were deposited on slides upon which the sequential reactions and washing steps were done automatically by the SOLiD™ 3 instrument. DNA attached to beads was sequenced with 50 rounds of oligonucleotide annealing and ligation (five different primers, 10 sequential ligations per primer). During each round, an oligonucleotide labeled with a fluorescent dye was annealed and ligated to the 3' end of the primer or the oligo ligated in the previous round. The next two unoccupied nucleotides of the strand attached to the bead determined which oligo ("color") was ligated. Use of staggered primers permitted determination of the unique base sequences. BioScope (version 1.2, Applied Biosystems, CA) was used to map the color sequences of each bead to the annotated FA1090 reference genome obtained from the National Center for Biotechnology Information (NCBI). Sequences were counted if they matched a 25-color end of a reference sequence with no more than 2 mismatches (permitting some mismatches is necessary because of sequencing errors and actual variations from the reference sequence). The length of the match was then determined by extending the alignment through the full 50-color sequence, allowing for more mismatches with increasing length. If a sequence read aligned to more than one genomic locus, the best alignment was selected. If a sequence read aligned equally well to more than one genomic locus, it was discarded. Stringency was set so that any base in the gonococcal genome had to be counted at least 10 times in order to be considered mapped. After mapping, colors were converted to bases. Sequencing data can be accessed by Gene Expression Omnibus (GEO; http://www.ncbi.nlm.nih.gov/projects/geo/) with the accession number GSE26444.

### Analysis of raw data

The Integrative Genomics Viewer (IGV) was utilized http://www.broadinstitute.com/igv to visualize raw sequencing data. The .wig files generated from RNA sequencing were loaded and compared against the gonococcal genome, allowing visualization of sequencing reads mapped outside of the annotated genome. This allowed for scanning of intergenic regions for detection of small RNAs and transcription start sites of genes.

### PCR

Genomic DNA from gonococcal strain F62 was isolated for use as a PCR template. Promoter sequences for *lac*Z fusions were amplified with iProof™ High Fidelity Polymerase (Bio-Rad, Hercules, CA). Clones were screened by PCR for presence and orientation of the insert using Amplitaq^® ^(Applied Biosystems, Foster City, CA). Primers available upon request

### Construction of *lac*Z fusions

Translational and transcriptional *lac*Z fusions were constructed in pLES94 [90]. Genomic DNA from gonococcal strain F62 was used as template. PCR fragments and pLES94 were cut with *BamHI*. Digested insert and plasmid were ligated and cloned into *E. coli *DH10B. Transformants were selected on LB medium plates containing chloramphenicol at 25 μg ml^-1 ^and 5-bromo-4-chloro-3-indolyl-ß-D-galactopyranoside (X-gal, Invitrogen) at 40 μg ml^-1^. Plasmids were checked for the presence and orientation of the insert by PCR, and those plasmids that contained an insert in the correct orientation were used to transform F62. Colony PCR was performed on chloramphenicol resistant colonies to confirm the presence of the reporter construct. The PCR product was also sequenced to ensure the appropriate fusion was made.

### Gonococcal transformation

Gonococci were transformed naturally. A light suspension of piliated cells was prepared in 1 ml of GCK broth containing 0.042% NaHCO_3 _and 10 mM MgCl_2 _[89]. Purified plasmid DNA was added and 100 μl of the suspension was plated on two GCK plates and incubated 6-9 hours at 37°C. Cells were then harvested from the plates and streaked on GCK plates containing chloramphenicol for selection of clones. Clones typically took 2 days to become visible on antibiotic plates.

### ß-Galactosidase assays

Gene reporter activity was determined by ß-galactosidase assays from cultures grown aerobically or anaerobically with nitrite [91]. For gonococcal cultures, sterile swabs were used to harvest cells from overnight plate cultures and cells were resuspended in Z-buffer [91]. Cells were lysed with chloroform and 0.1% SDS and assayed as described [91]. Activity is reported in Miller units and the reported results are the average of at least three assays performed in duplicate from each day the cultures were grown.

### Oligonucleotide and DNA sequencing

All synthesized oligonucleotides were obtained from Invitrogen, and confirmatory DNA sequencing was performed at ACGT Inc. (Wheeling, IL).

### Molecular biology techniques

Cloning and PCR techniques were performed in accordance to standard protocols [92-94]. Plasmid preparations were obtained with a QIAprep miniprep kit, and DNA fragments were purified with QIAquick PCR Purification or QIAquick Gel Extraction kits (QIAGEN, CA).

## Authors' contributions

VI performed analysis of RNA-seq data, constructed and analyzed all translational and transcriptional fusions, performed RT-PCR, and wrote the initial draft. VC guided the project and reformed later drafts of the manuscript. Both authors read and approved the final manuscript.

## Supplementary Material

Additional file 1 **Supplementary Table S1 (.xls): 50 bp SOLiD RNA sequence reads mapped to the annotated FA1090 genome**. This table contains all of SOLiD sequence reads that were mapped to the gonococcal FA0190 genome in our 2 biological replicates, and also contains values normalized to RPKM. Click here for file

Additional file 2 **Supplementary Table S2 (.pdf): Strain Table**. This file contains a list of the gonococcal strains used in this study. Click here for file
