# Supplementary material for: Deep sequencing-based analysis of the anaerobic stimulon in Neisseria gonorrhoeae
Source: BMC Genomics. 2011 Jan 20;12:51. doi: 10.1186/1471-2164-12-51 (PMC3032703; doi:10.1186/1471-2164-12-51)
Supplement: Additional file 2 — Supplementary Table S2 (.pdf): Strain Table. This file contains a list of the gonococcal strains used in this study. [file 1471-2164-12-51-S2.PDF]

Supplementary Table 2. Bacterial strains used in this study.

Contructs	Relevant genotype or properties	Source or reference
Plasmids		
pLES94	promoterless *lacZ* vector; Ap^r, Cm^r	(Silver & Clark, 1995)
pLES941	pLES94/*oxiA* from -200 to +9, fused to *lacZ*	This Study
pVI112	pLES94/*clpB* from -426 to +21 fused to *lacZ*	This Study
pVI113	pLES94/*recN* from -357 to +21 fused to *lacZ*	This Study
pVI114	pLES94/*npd* from -383 to +120 fused to *lacZ*	This Study
pVI115	pLES94/*hecA* from -350 to +12 fused to *lacZ*	This Study
pVI116	pLES94/*grx3* from -393 to +9 fused to *lacZ*	This Study
pVI117	pLES94*bfrA* from -387 to +42 fused to *lacZ*	This Study
pVI118	pLES94/*lexA* from -82 to +27 fused to *lacZ*	This Study
pVI119	pLES94/*fbpA* from -175 to +42 fused to *lacZ*	This Study
pVI120	pLES94/*fnrS* from -120 to -1 with *lacZ* leader region (included in primer) fused to *lacZ*	This Study
pVI121	pLES94/*fnrS* from -120 to +111 with *lacZ* leader region (included in primer) fused to *lacZ*	This Study
pVI122	pLES94/*fnrS* from -120 to +9 fused to *lacZ*	This Study
E. coli strains		
DH10B	Δ*lac*, Δ*endA1*, Δ*recA1*	Laboratory Collection
N. gonorrhoeae strains		
F62	pro^-	Laboratory Collection
RUG7900	F62 transformed with pLES941	This Study
RUG7901	F62 transformed with pVI112	This Study
RUG7902	F62 transformed with pVI113	This Study
RUG7903	F62 transformed with pVI114	This Study
RUG7904	F62 transformed with pVI115	This Study
RUG7905	F62 transformed with pVI116	This Study
RUG7906	F62 transformed with pVI117	This Study
RUG7907	F62 transformed with pVI118	This Study
RUG7910	F62 transformed with pVI119	This Study
RUG7911	F62 transformed with pVI120	This Study
RUG7912	F62 transformed with pVI121	This Study
RUG7913	F62 transformed with pVI122	This Study
